# Successful Management of a Refractory Orbital Pseudocyst in a Chinese Goose Using Intraorbital Gentamicin Injection

**DOI:** 10.1111/vop.70101

**Published:** 2025-10-15

**Authors:** Kristopher Houston, Chloe Fontaine, Natalia Dziedzic‐Nyrka, David Donaldson

**Affiliations:** ^1^ Hospital for Small Animals Royal (Dick) School of Veterinary Studies, University of Edinburgh Roslin Scotland UK

**Keywords:** gentamicin, goose, intraorbital cyst, orbital pseudocyst

## Abstract

**Objective:**

To report the successful management of recurrent orbital swelling in a Chinese goose ( *Anser cygnoides* ) following evisceration surgery, utilizing intraorbital gentamicin injection after multiple unsuccessful interventions.

**Animal Studied:**

A 3‐year‐old, 4.58 kg male Chinese goose ( *Anser cygnoides* ).

**Procedures:**

The goose was referred for evaluation of recurrent swelling of the right orbit following evisceration surgery performed after a severe corneal injury. Initial interventions included fluid drainage, revision surgeries with placement of Lyostypt and bone cement implants, sinus communication attempts, and placement of a Jackson‐Pratt surgical drain. Diagnostic evaluation included physical examination, computed tomography (CT), cytology, histopathology, and bacterial culture. Despite these interventions, the orbital swelling recurred. Finally, an intraorbital injection of gentamicin (9 mg/kg) was administered following fluid drainage. Post‐injection follow‐up was conducted over 6 months.

**Results:**

Following the intraorbital gentamicin injection, the recurrent swelling resolved without any adverse effects. The goose remained clinically normal at a six‐month follow‐up, with no signs of recurrence.

**Conclusions:**

This case highlights the challenges of managing recurrent orbital swelling in avian species following evisceration surgery and the potential utility of intraorbital gentamicin injections. Gentamicin's cytotoxic effects on secretory tissue may offer an effective treatment option for refractory cases. Further studies are warranted to explore the safety and efficacy of this approach in avian species.

## 1 Introduction

Orbital swelling following evisceration has been reported as a complication in avian species and poses a significant clinical challenge [1, 2, 3]. Modified evisceration, compared to enucleation, has been suggested as a preferred surgical technique to remove painful, non‐treatable globes in birds of prey [2]. Evisceration reportedly limits traction on the short optic nerve, avoids iatrogenic damage to the thin interorbital septum and fragile posterior orbital wall, and preserves facial symmetry [2]. Postoperative complications, similar to those described in mammals, include infection, hemorrhage, delayed wound healing, or dehiscence, and may also involve recurrent swelling or drainage caused by infection, retained epithelial or glandular tissue, seroma, or rare conditions like orbital emphysema [4]. This case report describes the management of a 3‐year‐old male Chinese goose ( *Anser cygnoides* ) referred for evaluation and treatment of chronic orbital swelling following evisceration surgery. It aims to present treatment options for captive birds experiencing postoperative orbital swelling after evisceration. The report highlights the use of intraorbital gentamicin injection, a technique previously used to ablate an intraorbital cyst in a duck with iatrogenic ankyloblepharon [1]. It discusses the challenges of managing this condition, the rationale for treatment, and the outcomes observed over a six‐month follow‐up period.

## 2 Materials and Methods

### 2.1 Case Presentation and Pre—Referral Management

A 3‐year‐old, 4.58 kg male Chinese goose ( *Anser cygnoides* ) was referred for evaluation of recurrent swelling of the right orbit following evisceration surgery. The initial evisceration was performed by the referring veterinarian to treat a corneal injury sustained during an altercation with another goose, but there was no confirmation of which specific tissues were removed. The procedure involved flushing the orbit and packing it with Lyostypt (B. Braun Medical Ltd., Sheffield, United Kingdom) before closing the subcutaneous and skin layers. Two weeks postoperatively, the skin over the right orbit became distended due to a fluid‐filled swelling.

One month post‐surgery, a revision surgery was performed, during which the wound was reopened, additional orbital contents were removed, and a Lyostypt was again placed prior to skin closure. The orbital swelling recurred within a month, and over the next several months, the orbit was drained of fluid percutaneously three times. Multiple courses of antibiotics were trialed unsuccessfully, including oral marbofloxacin, amoxicillin‐clavulanic acid, and enrofloxacin with no record of culture and sensitivity testing or the clinical reasoning that informed the antibiotic choices.

Six months after the first revision surgery, a second revision surgery was performed, during which a fenestration was drilled into the adjacent sinus to facilitate orbital drainage. Additional orbital tissue was removed, and the wound edges were trimmed before performing a two‐layer skin closure. However, fluid accumulation recurred within weeks.

During a third revision surgery, two poly methyl methacrylate (PMMA) beads were placed into the orbit to fill the dead space, and a 24‐gauge cannula was sutured in place to allow postoperative drainage. When the orbit filled with fluid again, the PMMA beads were removed, and a Lyostypt was placed into the orbit before closure. Anaerobic culture of a swab taken during this procedure isolated *Clostridium* spp. Aerobic cultures revealed moderate mixed growths of commensal flora, while yeast and fungal cultures were negative. At this stage, the goose was referred for further investigation and treatment. At the time of referral, the goose had been receiving oral meloxicam, tramadol, and a 3‐week course of oral clindamycin at 33 mg/kg twice daily.

### 2.2 Referral

**FIGURE 1:**
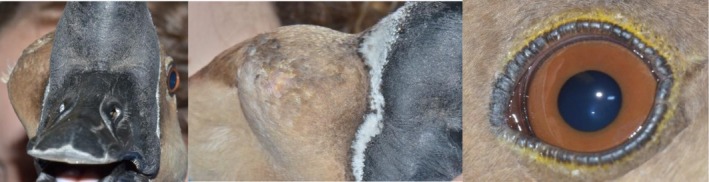
The goose on presentation showing a soft, depressible, non‐painful swelling over the right orbit (left and middle images) and an unremarkable left eye (right image).

The patient was referred to a specialist veterinary ophthalmology and exotics service 9 months after the initial evisceration surgery. On initial examination, marked subcutaneous swelling over the right orbital region was observed, as demonstrated in Figure 1. Palpation revealed the swelling was non‐painful, soft, and fluid‐filled, with no palpable fractures or discontinuities of the orbital rim. The left eye was examined using slit‐lamp biomicroscopy (Kowa Optimed SL‐17, Kowa Co, Japan) and indirect ophthalmoscopy (Keeler Vantage Plus, Windsor, UK) with a 90 diopter condensing lens (Volk Optical, Mentor, USA). The examination was unremarkable. Intraocular pressure (IOP) in the left eye was measured using rebound tonometry (TonoVet, iCare, Helsinki, Finland) and was presumed normal at 12 mmHg (normal: 9.1 to 11 mmHg in geese species [5]). The palpebral, dazzle, and direct pupillary light reflexes were present in the left eye.

### 2.3 Diagnostic Imaging

**FIGURE 2:**
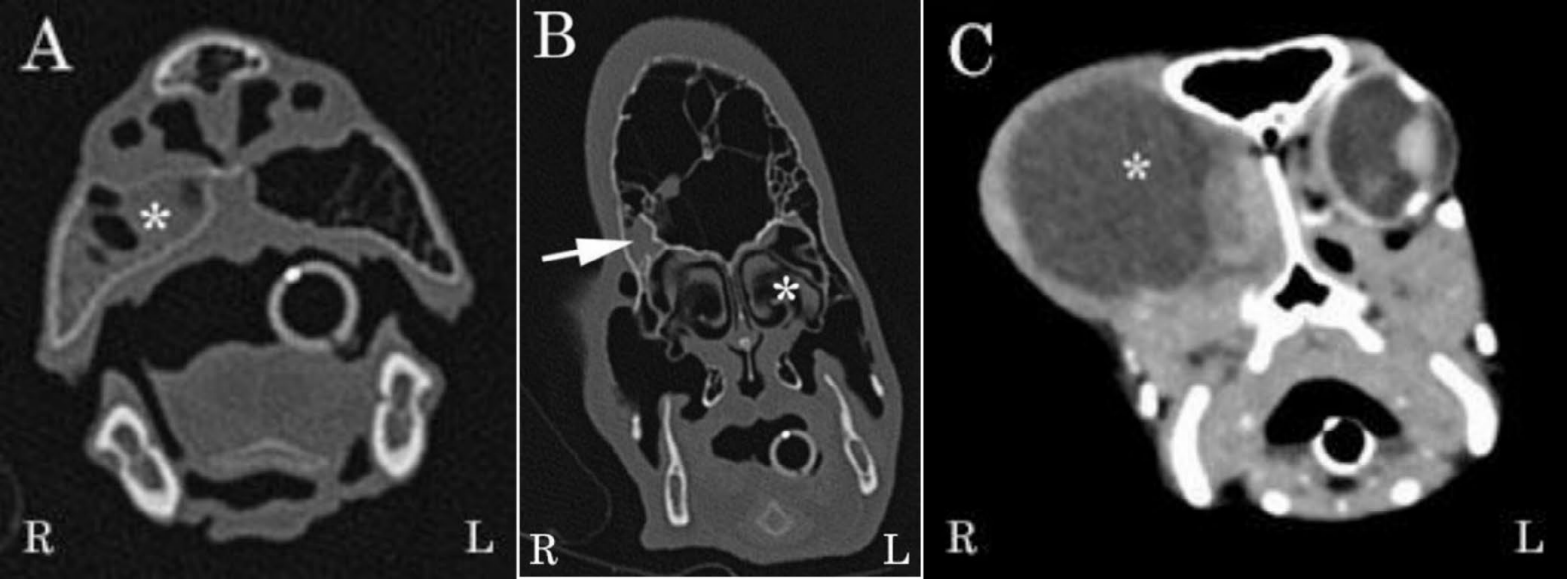
Coronal computed tomography (CT) images of the head highlighting the extensive sinus system and the orbits. (A) Bone window image illustrating a fluid attenuating content (asterisk) within the right premaxillary sinus. (B) Bone window image illustrating the nasal cavity (asterisk), paranasal sinuses, and extensive sinuses of the knob. The arrow indicates a fluid attenuating content in the right frontal sinus. (C) Soft tissue window post‐contrast image of the right orbit with a large, well‐defined, encapsulated fluid‐filled (asterisk) structure.

A full‐body computed tomography (CT) scan was performed under general anesthesia. The patient was induced with sevoflurane in oxygen delivered via a face mask and intubated with a 5.0 mm endotracheal tube. The CT scan revealed a large, round, well‐defined encapsulated fluid‐filled structure within the right orbit, presented in Figure 2. The right globe was absent, while the left globe appeared within normal limits. The encapsulated fluid‐filled structure within the right orbit was most likely a pseudocyst associated with scar tissue. Moderate gravity‐dependent fluid‐attenuating content was observed in the right premaxillary sinus and the ventral aspect of the right frontal sinus, with no evidence of bone destruction associated with the fluid accumulation. Mild right‐sided sinusitis and rhinitis were therefore diagnosed as a result.

### 2.4 Surgical Interventions

An exploratory surgery of the right orbit and drain placement was performed on the goose. Anesthesia was induced using sevoflurane gas in oxygen delivered via a facemask, followed by intubation with a 4.5 mm Portex endotracheal tube, which was cuffed for maintenance. Intravenous fluids were administered throughout the procedure at a rate of 10 mL/kg/h. During anesthesia, the goose was administered meloxicam (1.5 mg/kg subcutaneously) and amoxicillin‐clavulanic acid (125 mg/kg subcutaneously).

**FIGURE 3:**
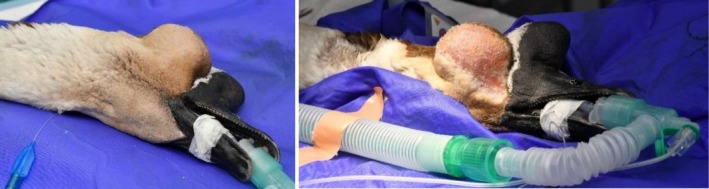
Preoperative appearance of the orbit (left) and the site after aseptic preparation (right).

The feathers over the right orbit were plucked, and the skin was aseptically prepared for surgery, as demonstrated in Figure 3. A small area of skin on the right side of the neck was also prepared as a precaution in case intraoperative arterial bleeding necessitated permanent carotid artery ligation [6]. An elliptical incision was made around the fibrotic wound from previous surgeries, and the skin was undermined using sharp and blunt dissection with Stevens tenotomy scissors. An encapsulated, fluid‐filled cavity beneath the skin was incised, and serosanguinous fluid was released from the orbit. After drainage, multifocal white, coliform plaques were observed on the orbital wall. A sample of orbital fluid was collected for cytology, and a swab of the plaques was submitted for bacterial culture and sensitivity testing.

**FIGURE 4:**
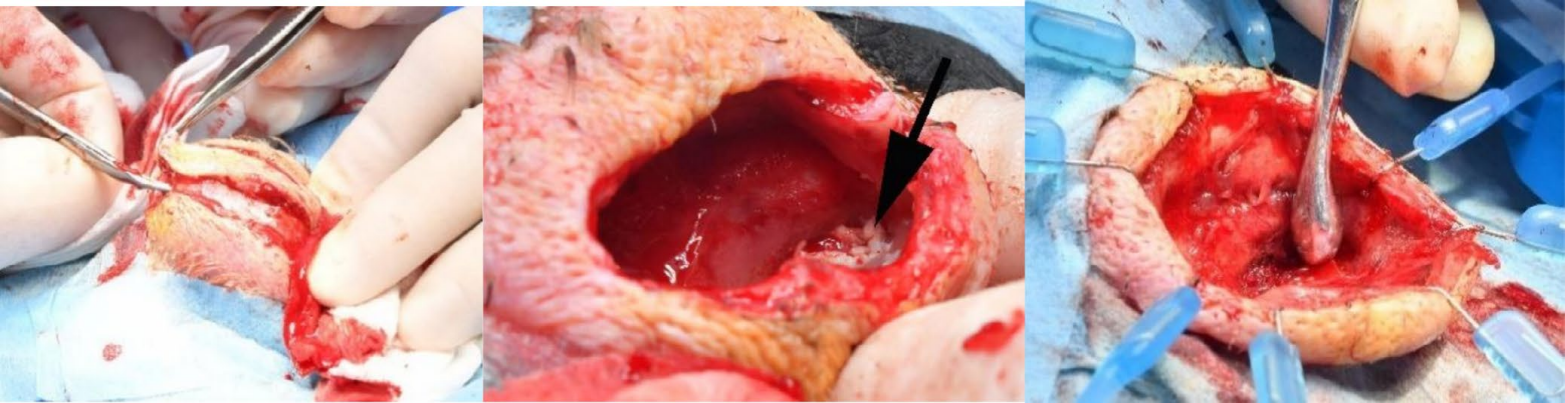
Perioperative steps, including dissection of the skin (left), orbital coliform plaques (arrow in middle image), and retraction of the wound edges using the Lone Star surgical retractor and debridement of the thin sheet of membranous tissue lining the orbital space (right).

**FIGURE 5:**
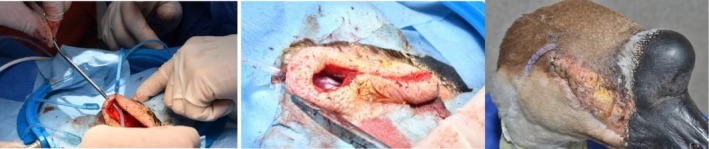
Perioperative drain placement (left), incision site closure (middle), and postoperative appearance (right).

Improved visualization and access to the orbit were achieved using a Lone Star surgical retractor (iM3, Northhampton, United Kingdom), illustrated in Figure 4. The orbital space was lined by a smooth thin sheet of membranous tissue, which was removed using curettage. Samples of the orbital wall lining were obtained for histopathological examination. No communication between the orbit and the sinuses was identified. The orbit was flushed with sterile saline, and a Jackson‐Pratt active surgical drain was placed within the orbit, exiting through the dorsocaudal orbital skin, demonstrated in Figure 5. The drain was secured in place with a finger trap suture and anchored around the neck using Soffban and Vetrap bandaging. The orbital wound was closed in three layers: A simple continuous suture pattern for the deep fascia and subcutaneous layers, and an intradermal pattern for the skin, using 4–0 Monocryl with a reverse cutting needle. Dermabond surgical adhesive was applied over the wound. The infraorbital sinus was accessed via an incision just caudal to the beak. The sinus lining appeared normal, containing a small amount of serous fluid, but no caseous material was observed. The skin incision was closed with several horizontal mattress sutures using 4–0 Monocryl and Dermabond surgical adhesive.

## 3 Results

### 3.1 Sample Analysis

Cytology of the orbital fluid revealed a predominantly mixed inflammatory response, with moderate numbers of heterophils admixed with low numbers of macrophages and the occasional small lymphocyte. Numerous bacteria were seen in the background, and rarely within heterophils, comprising a rather monomorphic population of coccobacilli.

The orbital wall was also sampled for cytology, which showed chronic mixed inflammatory cells with no evidence of epithelial or glandular tissue. Histopathology of samples taken from the orbital lining revealed multifocal pyogranulomatous folliculitis and dermatitis, with no epithelial or glandular tissue identified.

Swabs of the coliform plaques identified *Enterococcus* spp., which were sensitive to benzylpenicillin, enrofloxacin, marbofloxacin, doxycycline, tetracycline, nitrofurantoin, and chloramphenicol, but resistant to erythromycin. The results of routine biochemistry and hematology were unremarkable.

### 3.2 Postoperative Management

The patient tolerated the drain for 5 days following placement. On the first day, 17.6 mL of fluid was drained, and on the second day, 16.5 mL was removed.

On the third day, the patient traumatized the tubing, causing a loss of negative pressure, and the tubing required repair. On this day, only 6 mL of fluid was collected from the drain. The following day, 4 mL of fluid was collected. By the fourth day of hospitalization, the patient was no longer tolerating the drain, and it was removed. A total of 13 mL of fluid was collected prior to drain removal. Given the presence of coccobacillary bacteria on cytology (*Enterococcus* spp. isolated), oral amoxicillin‐clavulanic acid was prescribed for 7 days (at 110 mg/kg twice daily). Analgesia and anti‐inflammatory therapy consisted of 1 week of oral meloxicam (at 1 mg/kg once daily).

### 3.3 Intraorbital Gentamicin Injection and Repeat Fluid Analysis

The orbit began to swell again immediately following the removal of the surgical drain. The goose remained otherwise well, maintaining a normal appetite and exhibiting normal behavior. Three weeks after discharge and drain removal, the goose was re‐admitted for drainage of fluid and administration of a gentamicin injection into the orbit. The goose was sedated with 1 mg/kg intranasal midazolam. The skin over the right orbit was aseptically prepared, and a 5% lidocaine cream was applied. Twenty‐five milliliters of fluid were drained from the right orbit, after which 1 mL of 40 mg/mL gentamicin (9 mg/kg) was injected into the orbit. The goose recovered smoothly from the sedation and was discharged the same day. Cytology was repeated on the orbital fluid that had refilled the orbit. The fluid was hypocellular and contained foamy macrophages with erythrocyte breakdown products, consistent with aspiration of cystic fluid. There was no evidence of significant inflammation, microorganisms, or neoplasia. Repeated bacterial and fungal culture and sensitivity testing of the fluid was negative.

### 3.4 Long‐Term Outcome

**FIGURE 6:**
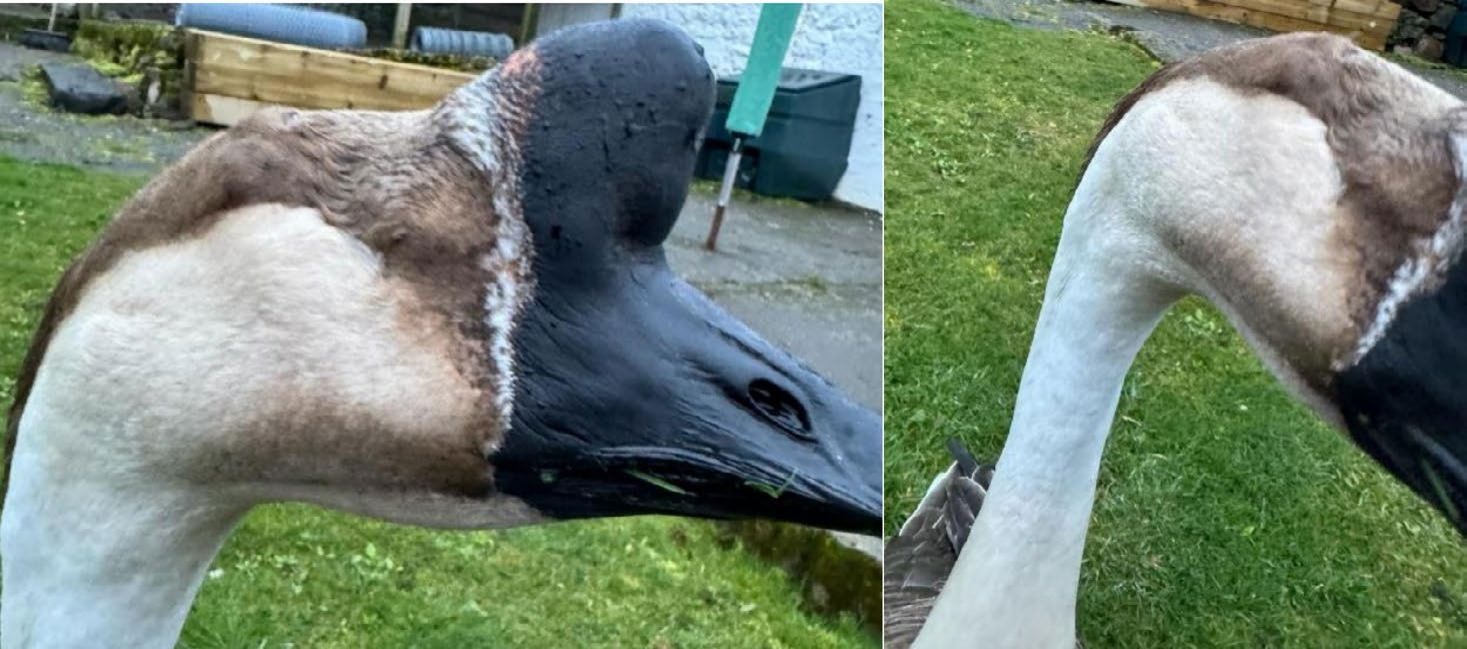
Images of the goose 6 months post intraorbital gentamicin injection.

At the six‐month follow‐up, the owner reported no recurrence of orbital swelling or clinical signs of infection, as seen in Figure 6. The goose displayed normal behavior and eating patterns, with no visible adverse effects from the gentamicin injection. This outcome confirmed the success of the treatment in resolving the refractory orbital pseudocyst and associated complications.

## 4 Discussion

Recurrent orbital cystic or seromatous complications following evisceration in avian species is a complex and challenging post‐operative complication [4], potentially influenced by the unique anatomical and physiological characteristics of birds, including specialized glandular tissues involved in tear secretion, such as the Harderian gland, lacrimal glands, and conjunctival tissue [7, 8]. This case report describes the first published successful management of a refractory orbital pseudocyst in a Chinese goose using an intraorbital gentamicin injection, highlighting an effective therapeutic approach. A similar technique was previously described in a duck, where it was used to treat an intraorbital cyst [1].

Orbital pseudocyst, rather than orbital cyst, was considered the most accurate description for the condition in this case, as no residual conjunctival or glandular tissue was identified on surgical exploration or histological examination of the orbital lining. Despite this, the continued production of large volumes of hypocellular fluid following repeated interventions suggested the possible presence of undetected secretory tissue, such as remnants of lacrimal or Harderian glands. Other avian case reports have described recurrent orbital swelling due to residual glandular tissue post‐surgery, resulting in the formation of an intraorbital cyst [1], or the development of orbital emphysema resulting from damage to the nasolacrimal duct [3].

Computed tomography has proven important in evaluating the pathology of the complex anatomy of the avian skull, defining the involvement of the nasal cavity, sinuses, and paranasal sinuses [9]. In this case, CT provided detailed visualization of the encapsulated fluid‐filled space expanding from the right orbit and associated changes in adjacent structures that were consistent with compression rather than bone destruction. Small amounts of attenuating fluid were also identified in the right premaxillary and right frontal sinuses with no direct communication with the orbital space, a finding that was subsequently confirmed at surgery.

Repeated bacterial and fungal culture and sensitivity testing of the fluid was negative.

The chronicity of the condition in this case was primarily attributed to the presence of residual secretory tissue, which likely sustained ongoing fluid production despite multiple surgical interventions. Although cultures identified multidrug‐resistant *Clostridium* spp. and *Enterococcus* spp., the lack of response to targeted systemic antimicrobials suggests that infection played a minimal role. Furthermore, the fluid aspirated from the orbit prior to gentamycin injection was hypocellular and consistent with the aspiration of cystic fluid with no evidence of significant inflammation, and repeated bacterial and fungal culture of the fluid was negative.

Conventional surgical approaches to manage seromas include debridement, the placement of drainage systems, and multiple courses of systemic antimicrobials [10]. These were insufficient to resolve the issue, underscoring the difficulty in managing cases of refractory postoperative orbital fluid accumulation [10].

Intraoperatively, the goose's neck was prepped in case permanent carotid artery ligation was required. Carotid artery ligation in birds is typically considered a salvage procedure in the event of uncontrollable hemorrhage and would be permanent rather than temporary. In chickens, bilateral carotid and vertebral artery ligation resulted in minimal behavioral changes and moderate weight loss in some individuals, with survival closely linked to individual vascular variability ([6]).

The decision to trial intraorbital gentamicin was guided by the literature [1, 11, 12, 13, 14]. The aim was to deliver a safe, therapeutic intervention targeting suspected retained glandular and/or epithelial tissues within the orbit. Gentamicin has known cytotoxic effects [11, 12], including cytotoxic effects on epithelial cells. There have been reports of retinal and choroidal toxic effects in humans and animals following intravitreal gentamicin injections [11, 13]. This technique has therefore been used as a therapeutic tool in end‐stage glaucoma to target the ciliary body epithelium, resulting in decreased aqueous humor production [13, 14]. In a report of a recurrent orbital cyst in a Rouen duck, Park et al. theorized that the use of an intralesional gentamicin injection successfully and safely resolved the cyst, potentially by destroying residual Harderian and/or lacrimal gland tissue [1].

Intraorbital polidocanol was also considered. Polidocanol is a sclerosing agent that has been used to treat cystic diseases such as bone, mucous, and renal cysts in humans [15]. The successful treatment of an orbital mucocele in a dog using 1% polidocanol [16] has been reported. However, the safety and efficacy of polidocanol in birds have not yet been reported. Gentamicin was chosen over polidocanol due to its established safety in avian species [17], an established systemic therapeutic dose range^18^and the prior success in a similar case [1].

A total dose of 20 mg (9 mg/kg) of gentamicin was chosen to balance efficacy and safety. This dose was previously successful in resolving an intraorbital cyst in a Rouen duck [1] and falls within established systemic avian therapeutic guidelines (5–40 mg/kg every 8–24 h) [18]. Following the injection, the goose in this study showed significant clinical improvement, and no immediate adverse effects were observed.

The resolution of the pseudocyst was not considered to be due to the antibacterial effects of gentamicin, for several reasons. Firstly, the large volumes of hypocellular fluid produced prior to the gentamicin injection suggested a secretory component to the disease process. Secondly, analysis of the fluid that refilled the orbit after the last surgical intervention was negative for both bacterial and fungal growth. Thirdly, no improvement had been observed with previous antibiotic trials, and the rapid resolution following a single treatment with gentamicin would not be expected in the context of a chronic, intractable infection. Finally, high levels of resistance to gentamicin among *Enterococcus* spp., a Gram‐positive, facultative anaerobic commensal, have been reported [19]. Given these considerations, it is more plausible that the resolution of the pseudocyst following gentamicin administration was, at least in part, due to its cytotoxic effect on epithelial cells [12] rather than its antimicrobial activity.

While the outcome in this case was favorable, further research is needed to evaluate the broader applicability of intraorbital gentamicin injections in avian species. The potential risks, including tissue necrosis and toxic nephropathies [20], warrant careful consideration and dosing adjustments based on species‐specific pharmacokinetics.

In conclusion, this report demonstrates the efficacy of intraorbital gentamicin injection in resolving a refractory orbital pseudocyst in a Chinese goose. This approach provides a viable alternative to repeated surgical interventions and expands the therapeutic options available for managing similar cases in avian patients.

## Disclosure

*Artificial Intelligence Statement*: The authors have not used AI to generate any part of the manuscript.

## Ethics Statement

As this is a clinical case report, ethical approval was not required. All treatment adhered to RCVS guidelines, with every effort made to minimize the patient's suffering.

## Conflicts of Interest

The authors declare no conflicts of interest.

## Data Availability

The data that support the findings of this study are available from the corresponding author upon reasonable request.
